# Screening for hemoglobin Bart’s disease among fetuses at risk at mid-pregnancy using the fetal cardiac diameter to biparietal diameter ratio

**DOI:** 10.1186/1471-2393-14-230

**Published:** 2014-07-16

**Authors:** Nopamas Thathan, Kuntharee Traisrisilp, Suchaya Luewan, Kasemsri Srisupundit, Fuanglada Tongprasert, Theera Tongsong

**Affiliations:** 1 Department of Obstetrics and Gynecology, Faculty of Medicine Chiang Mai University, Chiang Mai 50200, Thailand

**Keywords:** Spatio-temporal image correlation (STIC), 4D-ultrasound, Cardiac diameter, Biparietal diameter, Hb Bart’s disease, Homozygous alpha-thalassemia-1 disease

## Abstract

**Background:**

All sonomarkers used to screen for fetal hemoglobin (Hb) Bart’s disease need high expertise, preventing them from being widely used. Fetal cardiac diameter to biparietal diameter (C/B) ratio is a simple marker which has never been evaluated for its effectiveness. Therefore, we conducted this study to evaluate the effectiveness of C/B ratio in predicting fetal Hb Bart’s disease among fetuses at risk.

**Methods:**

Fetuses at risk of Hb Bart’s disease scheduled for diagnostic cordocentesis at 18 to 22 weeks of pregnancy were prospectively enrolled. All underwent ultrasound for fetal biometry and cardio-STIC acquisition for subsequent off-line analysis. Cardio-STIC volume datasets (VDS) were analyzed for cardiac diameter measurement and C/B ratio was calculated by the authors who did not know the fetal diagnosis. Final diagnosis of Hb Bart’s disease was based on fetal blood Hb typing.

**Results:**

Of 131 pregnancies enrolled to the study, 11 were excluded because of poor quality VDS. The remaining 120 were available for analysis. C/B ratio was significantly higher in the fetuses with Hb Bart’s disease than that in the unaffected ones (53.16% vs 41.68%, P < 0.001). C/B ratio could detect fetuses with Hb Bart’s disease with sensitivity of 91.5% and specificity of 77.6% (AUC ROC 0.929), using a cut-off point of greater than 45%.

**Conclusions:**

Among fetuses at risk, C/B ratio measurement at mid-pregnancy, using cut-off point of 45%, could effectively differentiate fetuses with Hb Bart’s disease from unaffected fetuses.

## Background

Hemoglobin (Hb) Bart’s disease or homozygous alpha-thalassemia-1 is the most common cause of hydrops fetalis in South East Asia and the disease is currently encountered increasingly in other parts of the world because of population migrations. In the area of high prevalence, prenatal control is commonly performed to avoid serious maternal complications secondary to hydrops fetalis. Ultrasound screening is an essential part of early prenatal diagnosis for Hb Bart’s disease before development of hydropic changes. Of ultrasound markers, cardiac diameter to thoracic diameter (C/T) ratio is most accurate in predicting the fetal disease [1,2]. In our practice, C/T ratio is the most common sonomarker used for predicting fetal Hb Bart’s disease. Though C/T ratio has a high accuracy as seen in several reports, it is often difficult for general practitioners who are not familiar with the fetal heart, since the criteria of proper C/T ratio require demonstration of symmetrical bilateral ribs in the same plane as the four-chamber view (FCV). Therefore, it seems to us that the accurate C/T ratio measurement is relatively difficult for general practice. Though such a screening should be performed by ultrasound specialists, general practitioners often have to do, especially in the rural areas with high prevalence of this disease. The main objective of this study was to develop a new simple and effective way to screen fetal Hb Bart’s disease as adjunct to standard anomaly screening, in the areas of high prevalence. Measurement of cardiac diameter to biparietal diameter (C/B) ratio is easier than C/T ratio. Since both biparietal diameter (BPD) and the FCV are included in routine anomaly screening. The extra-work needed is only adjustment of the FCV to be true FCV readily for measurement without worrying about the proper thoracic plane or symmetry of the fetal ribs as needed in C/T ratio measurement. The main objective of this study was to assess the accuracy of C/B ratio in predicting fetal Hb Bart’s at mid-pregnancy among fetuses at risk.

## Methods

A descriptive study of diagnostic test was undertaken at a tertiary center hospital, medical teaching school, with ethical approval of the institute review boards (Research Ethics Committee 4, Faculty of Medicine, Chiang Mai University, Study Code: OBG-13-1433-EX/Research ID 1433). Pregnancies at risk of fetal Hb Bart’s disease scheduled for prenatal diagnosis with cordocentesis at 18 to 21 weeks were enrolled to the study with written informed consent. A pregnancy at risk was confirmed when both of the couple were carriers of α-thalassemia-1 gene, diagnosed by PCR (SEA-type) technique. The pregnancies at risk were enrolled from our established screening program [3]. Gestational age was determined by fetal sonographic biometry in the first half of pregnancy. Exclusion criteria consisted of: 1) multifetal pregnancies; 2) fetal anomalies or chromosomal abnormalities; 3) fetal anemia due to any causes other than Hb Bart’s disease, and 4) unavailability of fetal diagnosis or final outcomes.

Before cordocentesis, a transabdominal 2D-ultrasound examination was performed for fetal anomaly screening and fetal biometry including biparietal diameter (BPD). In the same settings, Cardio-STIC volumes were acquired using a machine equipped with 2- to 5-MHz curvilinear transabdominal 3D-transducer Voluson E8 (GE Medical Systems, Zipf, Austria). The acquisition was performed on transverse plane of the fetal chest, starting with demonstration of typical 4-chamber views, both apical and transverse views, and followed by activating the transducer to transversely sweep through the fetal chest. The volumes were acquired when the fetuses were in quiescent state, with acquisition time ranging from 7.5 - 15 seconds and acquisition angles of 20–25 degrees depending on fetal cardiac size. All of the volume datasets (VDSs) were stored in the ultrasound machine’s hard drive for subsequent blindly off-line analysis by the authors who had no any information of the patients.

Fetal blood sampling via cordocentesis was carried out under transabdominal real-time ultrasound guidance using techniques as described elsewhere [4]. Definite fetal diagnosis of Hb Bart’s disease was based on cord blood analysis using high-performance liquid chromatography (HPLC).

**Figure 1:**
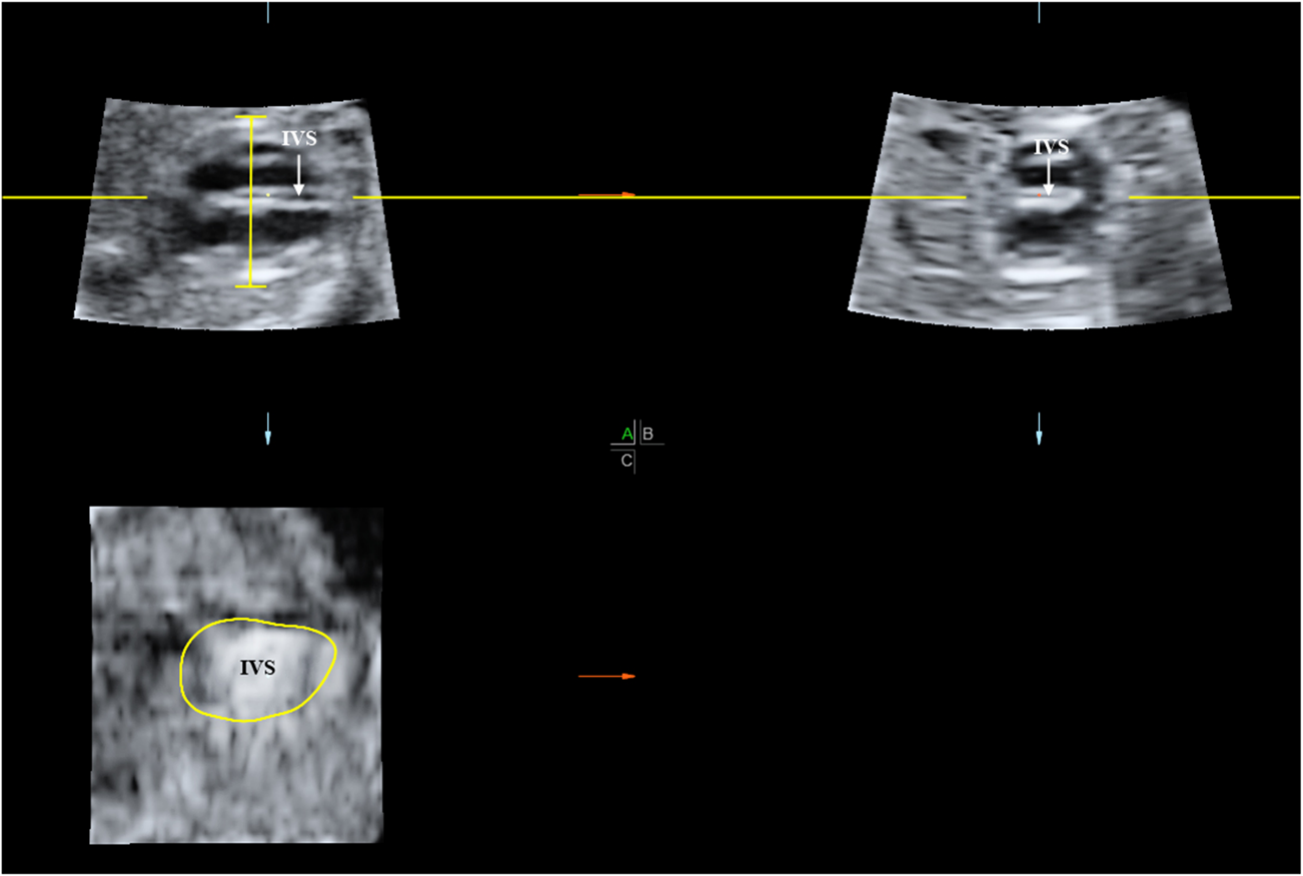
The plane for measurement of fetal cardiac diameter in the 4-chamber view at end diastole on multiplanar view after adjustiment of the cardio-STIC volume before displaying on a single full-screen image (IVS: interventricular septum).

Biparietal diameter (BPD) as a diagnostic parameter was relied on 2D-ultrasound measurement at the time of examination whereas fetal cardiac diameter was derived from off-line analysis of the cardio-STIC VDSs using 4D View processing software version 10.5 (GE Healthcare, Zipf, Austria). The cardiac diameter was biventricular outer dimension which was measured from the epicardium of the left ventricle to that of the right ventricle at the greatest dimension on typical FCV at end-diastole. To measure the cardiac diameter accurately, the VDSs were systematically adjusted to identify the true FCV as described elsewhere [5-7]. The true FCV in the panel A was then magnified and demonstrated in a full screen window. The proper image for measurement could be checked by demonstration that the interventricular septum in panel A was on the same line as in panel B and a total en face view of the interventricular septum (IVS) was visualized in panel C (Figure 1). The best measurements were selected and recorded for statistical analysis.

Based on previous studies of ultrasound sonomarkers in the second trimester [2], sensitivity of C/B ratio was expected to be approximately 94%. At a maximum allowable error of 0.1, and 95% confidence level, the study needed a sample size of at least 21 affected fetuses. The first separate 50 volume datasets were used to determine intra- and inter-observer variability and not included in analysis of the study.

### Statistical analysis

The stored data were analyzed for effectiveness of C/B ratio in predicting fetal Hb Bart’s disease, using the statistical package for the social sciences (SPSS) version 17.0 (Chicago, IL). The performance of C/B ratio in detecting the affected fetuses was assessed by sensitivity and specificity, negative and positive predictive value, using the best cut-off value based on the receiver operating characteristic (ROC) curve. P < 0.05 was considered statistically significant.

## Results

**Figure 2:**
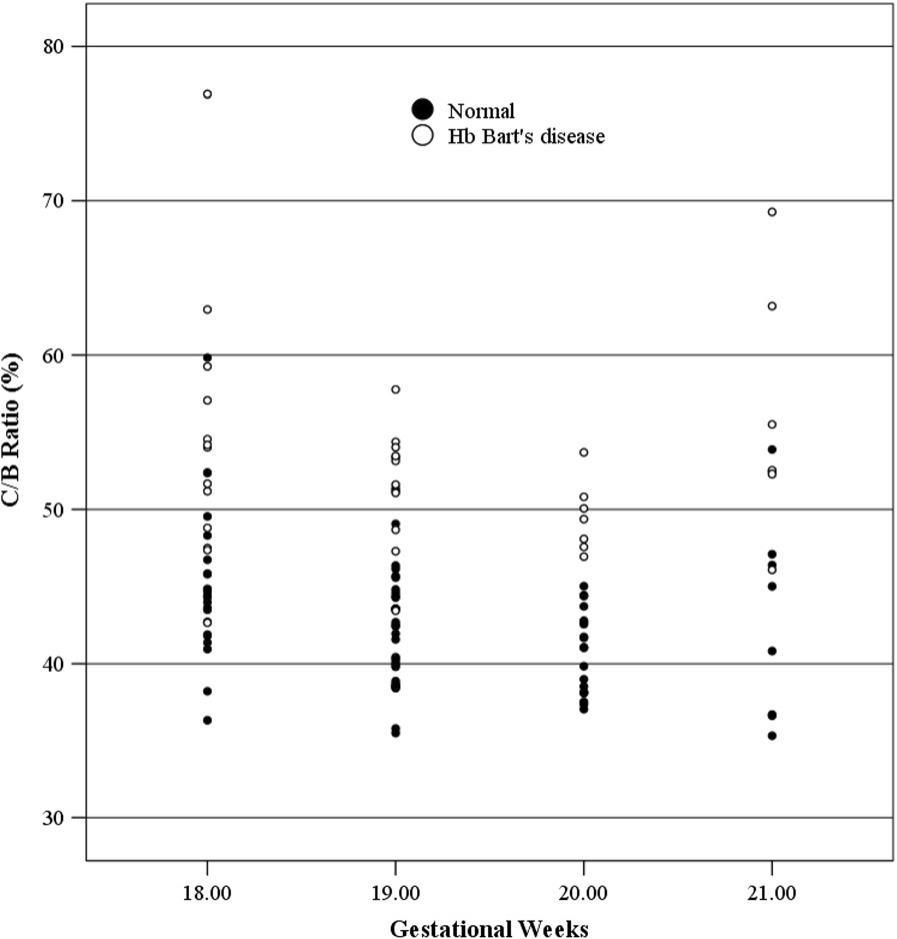
Scatter-plots of C/B ratio of normal fetuses and fetuses with Hb Bart’s disease for each gestational week.

**Figure 3:**
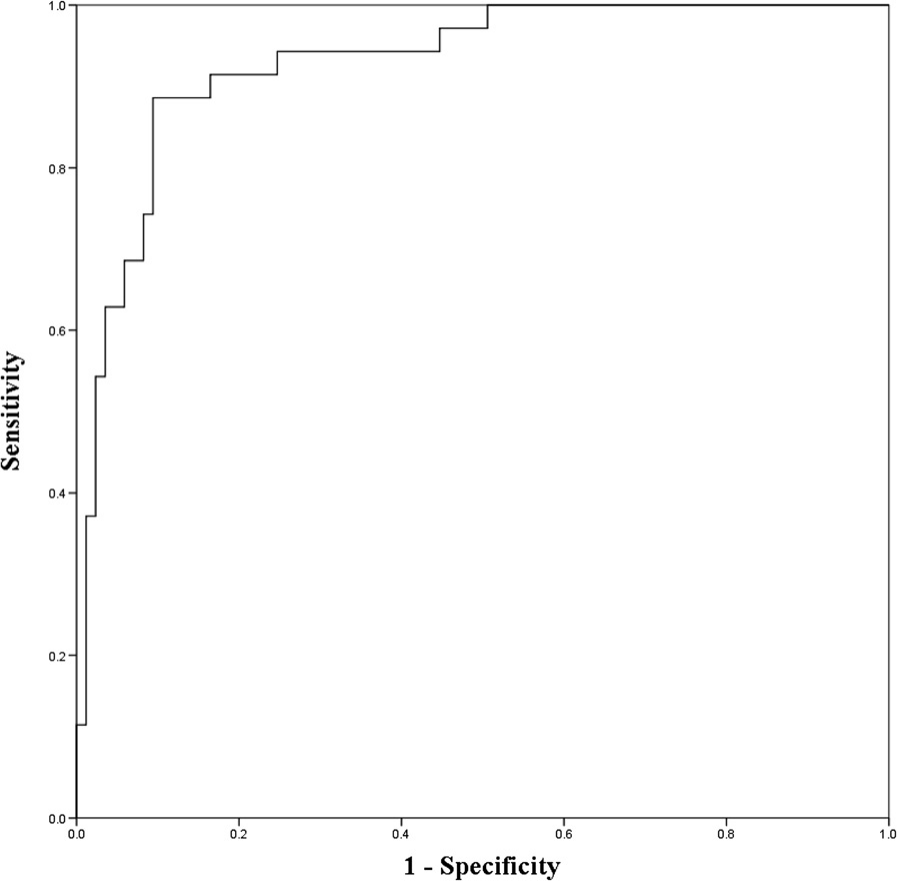
Receiver-operated characteristics (ROC) curve of the CB ratio in predicting Hb Bart’s disease (Area under curve 0.929).

Of 131 pregnancies enrolled to the study, 11 were excluded because of poor quality VDS due to obscured cardiac border. The remaining 120 were available for analysis. Mean gestational age at the time of ultrasound examination was 19.18 ± 0.99 weeks of gestation. Mean maternal age was 27.56 ± 6.35 years. Of all, 53% (62 women) were nulliparous and 47% (55) were multiparous. The C/B ratio was significantly higher in fetuses with Hb Bart’s disease than that in the unaffected fetuses (53.16% vs 41.68%, P < 0.001), as shown in Figure 2. Of interest, C/B ratio at mid-pregnancy (18–21 weeks of gestation) was relatively constant, not significantly changed with gestational age. Among unaffected fetuses, C/B ratios of fetuses at various gestational ages were not significantly different (P = 0.31). Likewise, distribution of the C/B ratios in affected fetuses was also not significantly changed with gestational ages, as presented in Table 1. The ROC curve for C/B ratio in detection of fetal Hb Bart’s disease had area under curve (AUC) of 0.929, giving the best cut-off value of 45%, as shown in Figure 3. Considering a cut-off value of 45% as an abnormal test, C/B ratio could detect fetuses with Hb Bart’s disease with a sensitivity of 91.5% and a specificity of 77.6%. The diagnostic indices of C/B ratio are presented in Table 2.

**Table 1:** Mean ± SD of cardiac diameter, biparietal diameter (BPD) and C/B ratio for each gestational week, according to affected and unaffected fetuses

**Gestational weeks**	**Number of cases (n)**	**Cardiac diameter (mm) (Mean ± SD)**		**P value**	**BPD (mm) (Mean ± SD)**		**P value**	**C/B ratio (Mean ± SD)**		**P value**
		**Unaffected group**	**Affected group**		**Unaffected group**	**Affected group**		**Unaffected group**	**Affected group**	
18	35	15.39 ± 1.27	17.67 ± 3.31	0.006	39.89 ± 1.06	38.68 ± 4.27	0.212	43.80 ± 4.84	53.46 ± 8.64	< 0.001
19	43	16.57 ± 1.59	19.04 ± 1.29	< 0.001	43.02 ± 1.30	43.38 ± 0.94	0.408	42.44 ± 3.93	51.64 ± 3.93	< 0.001
20	27	17.25 ± 1.26	20.25 ± 1.28	< 0.001	46.99 ± 0.77	46.48 ± 0.95	0.248	41.98 ± 2.86	49.35 ± 3.76	< 0.001
21	15	18.49 ± 0.96	21.11 ± 1.17	< 0.001	50.52 ± 3.54	48.77 ± 3.50	0.353	42.72 ± 6.48	53.89 ± 7.80	< 0.001

**Table 2:** Diagnostic indices of the C/B ratio in predicting Hb Bart’s disease (using 45% as a cut-off point; Area under curve 0.929)

	**Hb Bart’s disease**	**Normal**	**Total**
**Abnormal CB ratio**	32	19	51
**Normal CB ratio**	3	66	69
**Total**	35	85	120

Sensitivity: 91.43% (32/35) 95% CI: 82.20 - 100.7%.

Specificity: 77.65% (66/85) 95% CI: 68.80 - 86.5%.

Positive predictive accuracy: 62.70% (32/51) 95% CI: 49.50 – 76.00%.

Negative predictive accuracy: 95.770% (66/69) 95% CI: 90.1 - 101.2%.

Intra-observer variability in the measurements of C/B ratio showed intra-class correlation coefficient (ICC) 0.941 (95% CI: 0.916-0.958), Crombach’s alpha 0.969 and inter-observer variability ICC of 0.880 (95% CI: 0.828-0.916), Crombach’s alpha 0.910.

## Discussion

This is first study to assess the relationship between C/B ratio and fetal Hb Bart’s disease. We needed the most accurate measurements of the fetal cardiac diameters to identify such a relation. Therefore, we preferred using cardio-STIC, though it is currently not practical for widely use. If C/B ratio is a good predictor, we need further study to measure cardiac diameter with 2D-ultrasound in real practice to see whether it is reproducible or not. With conventional 2D-ultrasound, it is vulnerable to obtain an incorrect plane of the FCV, even in case that ultrasound beam is exactly perpendicular to the IVS, because it can be tilted toward or away from the performers on the z-axis, resulting in less accurate measurement of cardiac diameters. With cardio-STIC, IVS can simply be manipulated to get in a true horizontal line in panel A and B, while total en-face view of the IVS is displayed in panel C (Figure 1) [7]. The level of AV valves as used in some reports [8-10] may not be the best dimension for evaluation of cardiac size, since this is not the greatest transverse dimension. Therefore in this study the level of measurement was based on the maximal transverse dimension, just below the AV valve orifices as described by Sharland and Allan [11], which is easily demonstrated by adjustment of the VDSs. Biventricular outer diameter in the greatest dimension at end-diastole in this study was highly reproducible as indicated in analysis of inter- and intra-observer variability for intra-class correlation coefficient.

Based on this preliminary study, C/B ratio seems to be highly effective in predicting fetal Hb Bart’s disease among fetuses at risk, with a detection rate of 91.5% and specificity of 77.6%, using a cut-off point of greater than 45%. Additionally, C/B ratio at mid-pregnancy (18–21 weeks of gestation) was relatively constant, approximately 42% for normal fetuses. Therefore, it can probably be used as a date-independent parameter. The same cut-off 45% may be used in all gestational weeks at mid-pregnancies, leading to more convenience in general practice. While it has high accuracy like C/T ratio [2], its more simplicity and date-independency may make it more practical and easier to be widely used than conventional C/T ratio. This may lead to a new strategy of fetal anomaly screening at mid-pregnancy in the geographical areas of high prevalence of Hb Bart’s disease by adding C/B ratio measurement to the standard sonographic parameters. This may help us identify Hb Bart’s disease among the fetuses at risk at the time of routine fetal anomaly screening. Fetuses with abnormal C/B ratio can be offered definitive diagnosis by fetal blood analysis via cordocentesis, whereas those with normal C/B ratio can be offered serial ultrasounds instead of invasive diagnosis. With this approach several cordocenteces could be obviated. Nevertheless, the effectiveness of C/B ratio measured using 2D-ultrasound must be evaluated by further studies before implementation for real practice.

Though this study was based on fetuses with Hb Bart’s disease, it may theoretically be a model for sonographic study of fetal anemia secondary to other causes such as Rh isoimmunization or pavovirus-B19, etc. However, effectiveness in detection of fetal anemia using C/B ratio needs to be elucidated by further studies.

Some limitations of this study included as follows: 1) Though the objective of this study was to develop a new simple sonographic technique for widely use, it could not be applied for clinical use yet since the technique in this study was based on 3D-ultrasound STIC, further assessment of C/B ratio using 2D-real-time ultrasound has yet to be tested. 2) STIC has its unique limitations such as artifacts due to fetal movement during long acquisition (7.5-15 seconds). 3) The transducer of 2–5 MHz used in this study might be not optimal for cardiac scan in some cases. 4) BPD may theoretically be varied with fetal head shape, leading to unreliable C/B ratio, however in this study the mean BPDs of the affected and unaffected group were comparable, therefore, this could not affect the conclusion that the increased C/B ratio was reflexive of cardiomegaly rather than small BPD.

The strength of this study might include the followings: 1) adequate sample size to draw the conclusion, 2) highly homogeneous subjects, confined to fetuses at 18–21 weeks of gestations, the best time for fetal anomaly screening, 3) highly reliable methods of cardiac diameter measurements.

## Conclusions

Our results suggested that C/B ratio at mid-pregnancy, using a cut-off point of 45%, be effective, though not perfect, in differentiating fetuses with Hb Bart’s disease from unaffected ones.

## Abbreviations

4D-ultrasound: Four-dimension ultrasound; BPD: Biparietal diameter; Cardio-STIC: Cardio-spatio-temporal imaging correlation; C/B ratio: Cardiac diameter to biparietal diameter ratio; C/T ratio: Cardiac diameter to thoracic diameter (C/T) ratio; FCV: Four-chamber view; Hb Bart’s: Hemoglobin Bart’s; HPLC: High-performance liquid chromatography; PCR: Polymerase chain reaction; VDS: Volume datasets.

## Competing interests

The authors declare that they have no competing interests.

## Authors’ contributions

NT and TT conceived and designed the study. NT, KT and TT performed volume dataset analysis and statistical analysis, SL, KS, and FT performed cardio-STIC acquisition. NT and KT drafted the manuscript. TT supervised manuscript modification. All authors contributed to the interpretation and writing of the paper and approved the final version.

## Pre-publication history

The pre-publication history for this paper can be accessed here:

http://www.biomedcentral.com/1471-2393/14/230/prepub
